# Engaging citizens in the development of a health system performance assessment framework: a case study in Ireland

**DOI:** 10.1186/s12961-021-00798-8

**Published:** 2021-12-20

**Authors:** Óscar Brito Fernandes, Erica Barbazza, Damir Ivanković, Tessa Jansen, Niek S. Klazinga, Dionne S. Kringos

**Affiliations:** 1 Department of Health Economics, Corvinus University of Budapest, Fővám tér 8, 1093 Budapest, Hungary; 2 Department of Public and Occupational Health, Amsterdam UMC, Amsterdam Public Health research institute, University of Amsterdam, Meibergdreef 9, 1105 AZ Amsterdam, The Netherlands

**Keywords:** Public deliberation, Priority-setting, Focus group, Stakeholder panel, Healthcare system performance, Performance intelligence, Ireland, Sláintecare

## Abstract

**Background:**

The launch in 2017 of the Irish 10-year reform programme *Sláintecare* represents a key commitment in the future of the health system. An important component of the programme was the development of a health system performance assessment (HSPA) framework. In 2019, the Department of Health of Ireland (DoH) and Health Service Executive (HSE) commissioned the technical support of researchers to develop an outcome-oriented HSPA framework which should reflect the shared priorities of multiple stakeholders, including citizens. This study describes the method applied in the Irish context and reflects on the added value of using a citizen panel in the development of an HSPA framework.

**Methods:**

A panel of 15 citizens was convened, recruited by a third-party company using a sampling strategy to achieve a balanced mix representing the Irish society. Panellists received lay-language preparatory materials before the meeting. Panellists used a three-colour scheme to signal the importance of performance measures. An exit questionnaire was administered to understand how participants experienced being part of the panel. The citizen panel was the first in a series of three panels towards the development of the HSPA framework, followed by panels including representatives of the DoH and HSE, and representatives from professional associations and special interest groups.

**Results:**

The citizen panel generated 249 health performance measures ranging across 13 domains. Top-ranking domains to the citizen panel (people-centredness, coordination of care, and coverage) were less prioritized by the other panels; domains less prioritized by the citizen panel, such as accessibility, responsiveness, efficiency, and effectiveness, were of higher priority in the other panels. Citizen panellists shared a similar understanding of what a citizen panel involves and described their experience at the panel as enjoyable, interesting, and informative.

**Conclusions:**

The priorities of the citizen panel were accounted for during all phases of developing the HSPA framework. This was possible by adopting an inclusive development process and by engaging citizens early on. Citizen engagement in HSPA development is essential for realizing citizen-driven healthcare system performance and generating trust and ownership in performance intelligence. Future research could expand the use of citizen panels to assess, monitor, and report on the performance of healthcare systems.

**Supplementary Information:**

The online version contains supplementary material available at 10.1186/s12961-021-00798-8.

## Background

Health system performance measurement, and its use as performance intelligence, plays a central role in guiding the decisions of health system actors towards improved outcomes [1] and care experiences [2]. Publicly reporting performance measurement can also contribute to encouraging improvements [3], as well as system transparency and accountability [1, 4], the latter contributing to public trust and a sense of ownership of the healthcare system [5]. Of the varied approaches to measure performance [1], the use of health system performance assessment (HSPA) is a well-established and widely supported [6, 7] means to evaluate the health system as a whole. HSPA is a country-specific participatory process, relying on a limited number of indicators to link outcomes with system functions or strategies [8]. In the European context, the development of tools and methodologies to support performance measurement [9–11] and a range of country studies on the use of HSPA [12–15] have served to shift discussions from why use an HSPA approach to focus on the what and how of the process.

Citizen engagement processes can take on varied forms, such as citizen panels, focus groups, citizen juries, consensus conferences, or citizen assemblies [16, 17]. Importantly, these methods are distinct from more passive approaches focused on preference elicitation, such as polling and surveying. In contrast, citizen panels put emphasis on empowering citizens to actively deliberate in processes that precede policy-making [18]. This contribution is presumed to have important advantages in supporting ownership, legitimacy, and fairness in priority-setting processes, as well as building public trust and greater acceptance in decisions [5, 19].

In Ireland, improving the governance, accountability, and performance of the health system was set out as a key priority in the 10-year reform programme *Sláintecare* 2019–2028 [20]. At the outset of the programme, key stakeholders recognized that a comprehensive performance measurement framework and management system was needed to foster accountability and capture achievements against the objectives of *Sláintecare* [21]. To this end, the Department of Health of Ireland (DoH) requested the support of the European Commission’s Structural Reform Support Service, and in mid-2019, the project “Performance Accountability for the Irish Health System” was launched. A team of Healthcare Performance Intelligence Professionals from Amsterdam University Medical Centers was selected to support this project. Early in the project, priority was given to selecting a set of domains and performance measures that reflected shared priorities of multiple stakeholders, including citizens.

While methods for engaging stakeholders are well established in their own right [22, 23], citizen participation applied to HSPA processes has yet to be embedded into development cycles [15, 24]. The engagement of citizens in participatory policy processes such as the development of an HSPA framework links to the broader discussion on value-creating learning health systems [25]. Being considerate of the central role of citizens’ care experiences and perceptions about the healthcare system can strengthen value creation and nurture public trust and a sense of ownership [5], hence contributing to the healthcare system becoming more people-centred and prioritizing care and outcomes that matter most to patients [26]. For example, the International Consortium for Health Outcomes Measurement has recognized the importance of participatory processes by engaging patients and healthcare professionals in developing patient-reported outcome measures (e.g. [27]). Like these participatory processes, with this study we set out to apply methods for the engagement of both citizens and other stakeholders towards the development of Ireland’s first HSPA framework. From the project’s inception, we had planned for processes to engage stakeholders following fundamental design principles of stakeholder engagement (e.g., defining a clear aim of engaging stakeholders, planning interactions with stakeholders and how their input can be collated, analysed, and used, and fostering value of shared learnings across stakeholders) [28]. This study aims to describe the method applied in the Irish context to convene a citizen panel for the purpose of developing an HSPA framework, and to reflect on the added value of using a citizen panel in the development of an HSPA framework.

## Methods

### Design

**Fig. 1:**
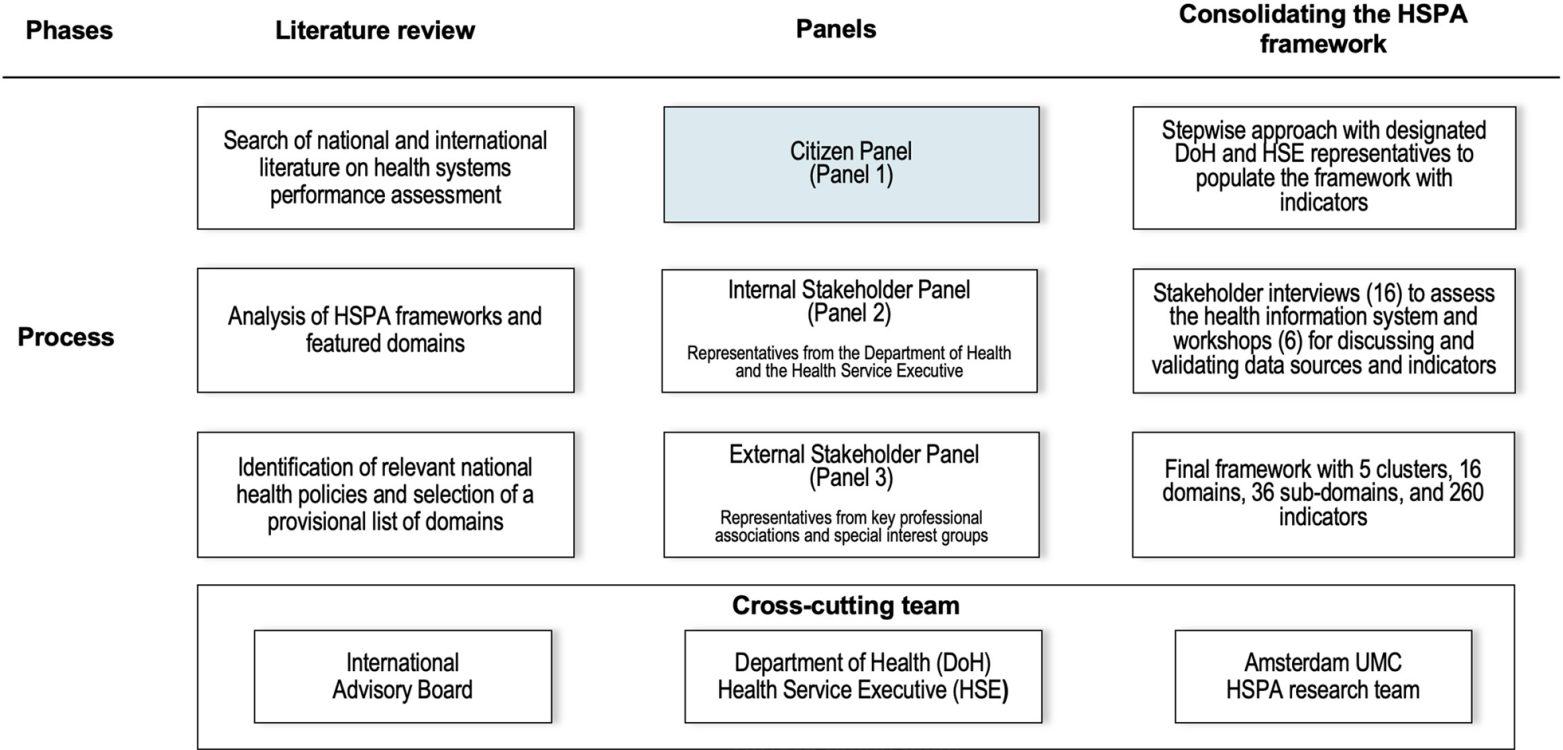
Processes and actors involved in the development of the HSPA framework for Ireland

This study focuses on the novelty of using a citizen panel for the purpose of developing an HSPA framework. The citizen panel (panel 1) was conducted in December 2019 and included 15 people of varying walks of life (Fig. 1). This panel was the first in a series of three panels towards the development of the HSPA framework. Panel 2 included representatives of the DoH and Health Service Executive (HSE) (internal stakeholder panel); panel 3 included representatives from key professional associations and special interest groups such as the academic and patient organizations (external stakeholder panel). Approximately 50 people identified by the DoH and HSE participated in panels 2 and 3 (30 people in panel 2 and 20 in panel 3), in January 2020. Additional information on how panels 2 and 3 were run is available as Additional file 1. Participants in the citizen panel were compensated for their travel and received a stipend for their time; they also signed an informed consent form prior to engaging in the meeting and were aware they could withdraw from participating in the meeting at any time.

### Literature review

At the outset of the study, a targeted review of the scientific and grey literature on HSPA was conducted to consolidate a list of potential domains for the framework being developed in Ireland. We took as a basis an international comparative study of HSPA domains applied across the WHO European Region’s 53 Member States [12], as well as an internal review by the DoH. The list of domains (health and well-being, equity, accessibility, quality of care, social and financial risk protection, coverage, safety, responsiveness, efficiency, effectiveness, people-centred, continuity of care, and coordination of care) and their definitions were consolidated for use in the panels that followed.

### Citizen panel

#### Sample and recruitment

A target of 15 panellists was pursued based on prior experiences with using citizen panels in the health sphere [29]. Citizens were recruited by a Dublin-based company providing recruitment services for qualitative research. The definition of a citizen eligible to participate in the panel was that of a layperson, resident of Ireland, who was not professionally involved with the healthcare system and was not a public official. The sampling strategy aimed to achieve diversity among participants, taking into consideration eight factors: representation of different counties; sex; age; highest level of education attained; nationality; ethnicity; health status; and religious beliefs. A recruitment scheme was developed based on key characteristics of the population according to 2016 census data [30] (Additional file 2). The recruitment agency reached prospective panellists by phone through an established registry. A screening questionnaire was applied, which informed citizens on the context of this study and assessed the diversity factors to ensure the targeted sample was achieved (Additional file 3).

#### Workshop

A lay-language citizen brief and workshop programme—both revised by the DoH for clarity of language—were prepared and shared with panellists via email 1 week before the workshop (Additional file 4). Two moderators (EB and ÓBF) led the panel’s work, and one notetaker (DK) took detailed notes throughout the day. A letter of consent was completed at the outset of the workshop. Participants were seated in a U shape and were able to interact with one another. The day’s activities were organized in three blocks. First, a round of introductions of both moderators and participants was conducted. We restated the basis for convening the citizen panel, as detailed in the citizen brief, to ensure the context was understood, together with the aims and organization of the panel. As a warm-up, the moderators asked participants about how satisfied they were with their healthcare system and how they got access to information about its performance.

Second, we conducted a prioritization exercise on what performance measures of Ireland’s healthcare system should be reported to the public. The moderators started off by summarizing the domains most frequently used in other countries to report on the performance of their healthcare systems. After the presentation of a domain, citizens were asked to write down on sticky notes what performance measures or performance-related topics were of concern to them. The colour scheme of sticky notes sought to capture the degree of importance assigned by a panellist to a performance measure/performance-related topic (red: extremely important; yellow: important; green: somewhat important). When all domains had been discussed, the moderators grouped the sticky notes on a whiteboard, producing a heat map. This mapping was used to clarify and confirm the panellists’ priorities in terms of the ranking of domains (gauged by the number of sticky notes) and the most frequent performance measures written by panellists. Participants discussed these results, often supporting their views with their personal experiences and those of their families and communities. Participants were also asked to identify approaches and channels whereby information on the healthcare system could be reported back to the public.

Third, participants were asked to complete an anonymous questionnaire asking (1) the panellist’s experience in taking part in the workshop; (2) how well prepared the panellist felt to take part in the panel; (3) the completion of a projective-inspired task [31] by completing the sentence “Citizen panels are…”; and (4) aspects related to the panellist’s experience or comments regarding the organization of the event (Additional file 5).

After the workshop, we prepared a summary of key findings of the citizen panel to share with participants (Additional file 6). The summary was sent to participants via email by the recruiting company. Citizen panel participants were encouraged to review and validate key findings in terms of accuracy, missed points, and last thoughts.

### Consolidating the HSPA framework

After the three stakeholder panels, we organized their ranking of domains and suggestions of performance measures and performance-related topics in a spreadsheet. Performance measures from the stakeholder panels were added to a pool of indicators that resulted from an indicator review of HSPA frameworks and existing standardized indicators of international organizations. We then followed a stepwise approach with designated DoH and HSE representatives to populate the framework with indicators. In parallel, we performed an assessment of the health information system in Ireland, which included 16 interviews with stakeholders identified by the DoH and HSE. Thereafter, six workshops were conducted with stakeholders for discussing and validating data sources and each indicator against fitness-for-purpose and fitness-for-use criteria [32]. Inputs from these workshops were consolidated to produce the final version of the HSPA framework for Ireland, which comprised five clusters, 16 domains, 36 subdomains, and 260 indicators (Additional file 7).

### Data analysis

The research team assigned the coloured sticky notes produced during the citizen panel to HSPA domains. Data from the prioritization exercise with the citizen panel were consolidated in a ranking chart. For the citizen panel, the ranking is based on the number of sticky notes participants produced per domain; for the internal and external stakeholder panels, the ranking is based on the number of times a domain was discussed by the participants of those panels. Thereafter, we compared the priority ranking of domains of the citizen panel relative to those consolidated during the internal and external stakeholder panels. These panels followed a similar structure to that of the citizen panel (Additional file 1). Content analysis was performed for the four-question exit questionnaire applied to the citizen panel participants. Quotes were selected to strengthen our interpretation of the data or to illustrate a participant’s experience during the panel. To improve the validity of the results, we triangulated data from multiple sources [33], including notes from the panel sessions, feedback from the moderators, experience sharing and discussions among the research team, and questionnaire data. The last was particularly valuable for synthesizing the experience of citizens during the panel, using their own words.

## Results

### Characteristics of the citizen panel participants

A total of 15 participants (7 men and 8 women) participated in the citizen panel (Table 1). The age group distribution was as follows (in years): 18–24 (*n* = 2); 25–34 (*n* = 4); 35–44 (*n* = 1); 45–54 (*n* = 4); 55–64 (*n* = 1); and 65 and over (*n* = 3). The highest educational level for four participant was junior certificate (primary education); four participants attained a leaving certificate (secondary education), six a bachelor diploma, and one participant had a graduate/master’s certification (tertiary education). Most participants had a paid job (*n* = 7), reported being in good health (*n* = 11) and lived in urban areas (*n* = 14). The nationality of most respondents was Irish (*n* = 13), including one of Asian descent, and two had nationalities of other European countries (Croatia and Italy) but resided in Ireland.

**Table 1:** Citizen panel participants’ characteristics

Characteristics of the panellists	*n*	%
Sex		
Female	8	53
Male	7	47
Age (years)		
18–24	2	13
25–34	4	27
35–44	1	7
45–54	4	27
55–64	1	7
65+	3	20
Education		
Primary	4	27
Secondary	4	27
Tertiary	7	47
Employment status		
With a paid job	7	47
Without a paid job	3	20
Student	2	13
Retired	3	20
Health status (good health)	11	73
Location (urban)	14	93
Nationality		
Irish	13	87
Italian	1	7
Croatian	1	7

### Citizen panel prioritization of HSPA domains

**Fig. 2:**
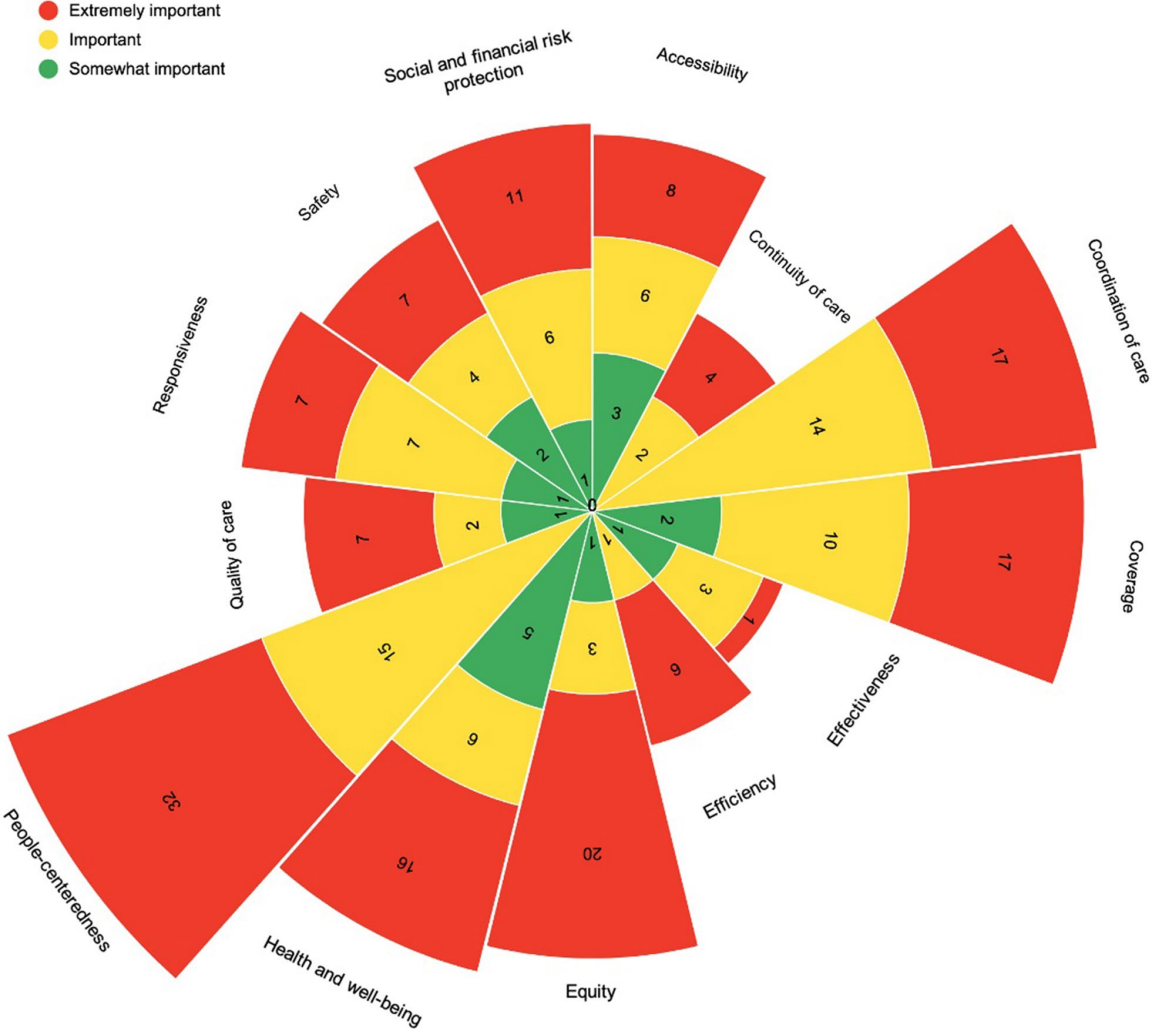
Citizen panel prioritization of HSPA domains with breakdown by degree of importance

During the panel session, 249 coloured sticky notes were used by participants with suggestions of health system performance measures or performance-related topics (including potential duplicates, i.e., different participants could have suggested similar performance measures): 153 were marked as extremely important, 79 as important, and 17 as somewhat important (Fig. 2). The domains with the most performance measures proposed were people-centredness (*n* = 47), coordination of care (*n* = 31), coverage (*n* = 29), health and well-being (*n* = 27), and equity (*n* = 24); within these, most of the measures were considered “extremely important”. The performance measures and performance-related topics suggested by panellists are available as supplementary material (Additional file 8). We received no reactions from participants regarding the citizen panel summary prepared with key findings of the panel.

**Fig. 3:**
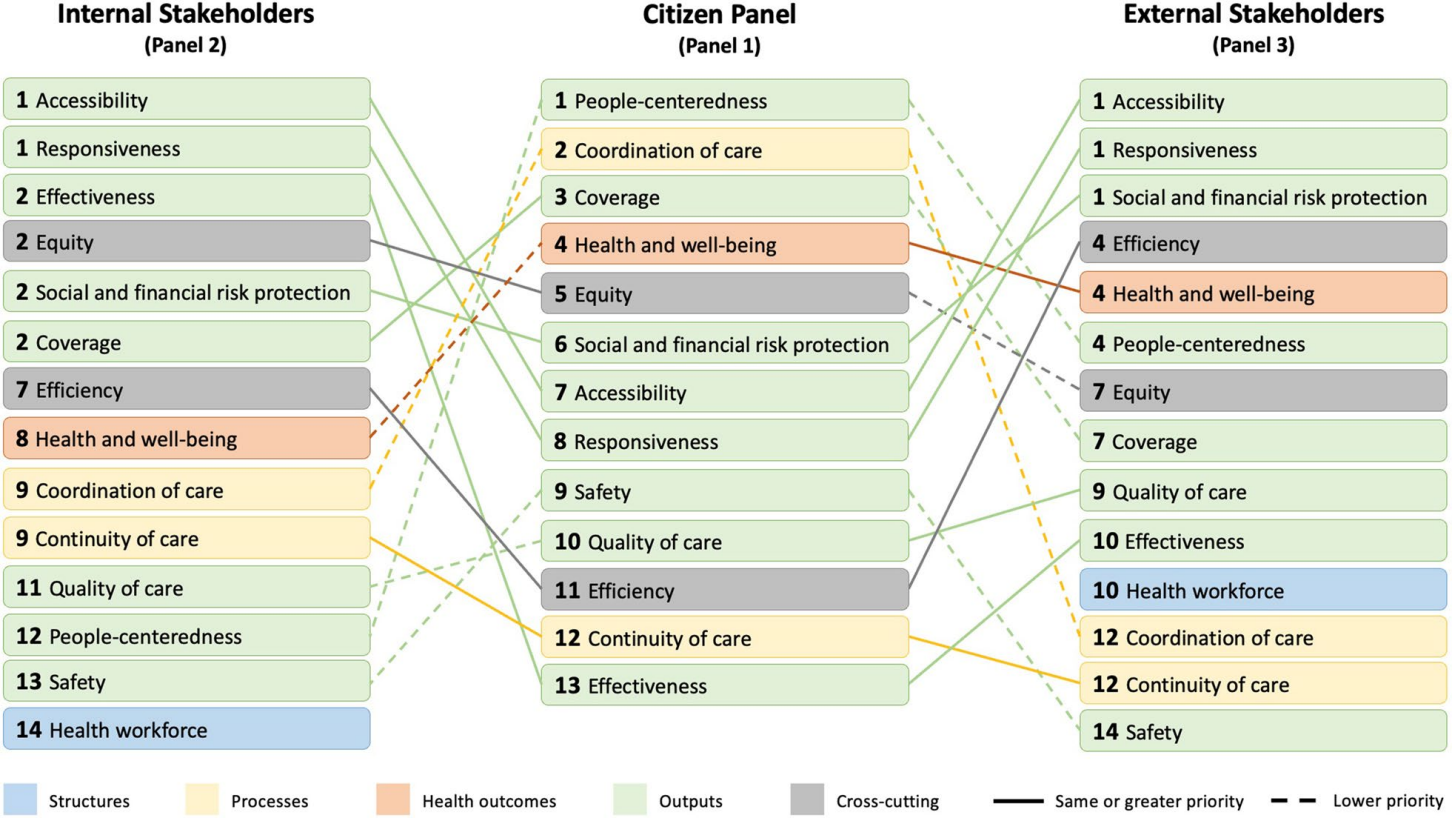
Prioritization of HSPA domains by the citizen panel relative to the other stakeholder panels. Colour indicates clustering of domains as depicted in the produced HSPA framework for Ireland

The internal stakeholder panel (panel 2) considered accessibility and responsiveness as being top-priority domains for the Irish HSPA framework, followed by effectiveness, equity, social and financial risk protection, and coverage as a second-tier priority (Fig. 3). Similar top priorities were observed in the external stakeholder panel (panel 3), with accessibility, responsiveness, and social and financial risk protection being considered top-priority domains. We observed great variation in the priority assigned to each domain by the citizen panel compared to the prioritizations assigned by the internal and external stakeholder panels. In most instances, the top-ranking domains for the citizen panel (people-centredness, coordination of care, and coverage) were less prioritized by the other panels; domains that were less prioritized by the citizen panel, such as accessibility, responsiveness, efficiency, and effectiveness, were of higher priority in the other panels. “Health workforce” was suggested as a stand-alone domain by both the internal and external stakeholder panels, whereas in the citizen panel the discussion was brief and centred on the inefficiencies of the workforce working environment (e.g., paper workload).

### Citizen panel participants’ experiences

Participants described their experience during the citizen panel as enjoyable, interesting, and informative. The citizen panel was depicted as an opportunity to discuss and develop a broader understanding of the functioning of Ireland’s healthcare system by the experiences and viewpoints of other participants, as highlighted by two of the panellists:

*I am more aware now on how the health system works through others*. (Panellist #15)

[Participating in the citizen panel was] *Very interesting and informative. Did not realise how difficult it was for people going through the public system. Hardship on public system seems much bigger that shared across social media*. (Panellist #3)

All participants appeared well prepared for the panel work; this was highlighted by the participants themselves, who mentioned that the citizen brief was sufficient for their preparation, as suggested by one participant: “*Not an issue with pre-reading materials and guidance of facilitators*” (Panellist #11). Four participants revealed that they relied on their experiences with the healthcare system to engage in the panel’s discussions, hence feeling confident of their preparation for the panel’s work, as highlighted by a panellist: “*[I felt] Very well prepared as the only thing requested was our own experience*” (Panellist #9). Other participants prepared themselves differently ahead of the citizen brief: “*Done quite a lot preparation in the past week, including to ask others on their views of the health service and what they thought to be important to improve it*” (Panellist #12). Participants also mentioned the importance of using lay language at all times during the panel session, including any written materials (e.g., the citizen brief), to keep participants engaged, and thus positively affect their experiences.

In general, participants showed similar social representations of what a citizen panel entails, describing it as “[a method of] *eliciting info from different ideas/experiences from a diverse group*” (Panellist #11) and “*…allowing debate to summarise and retain the opinions and ideas of the citizens into more actionable and basic concepts and thoughts*” (Panellist #5), which could be “*…very important for people to be able nonymously* [sic] *express their beliefs and talk about their experience in hope of a change for better*” (Panellist #9).

## Discussion

We described the methods of using a citizen panel as a key element of the development of an outcome-oriented HSPA framework for Ireland. The citizen panel prioritized more people-centredness aspects of performance domains compared to the internal and external stakeholder panels. This prioritization by the citizen panel is aligned with an interpretation of the Irish Health Survey 2019 data, which suggested the need for reform of the health system to achieve greater value, performance, and person-centred quality of care [34]. To achieve such goals and foster citizen-driven healthcare system performance, including citizens’ views is key in steering the health system to better address their needs, expectations, and preferences.

Citizen panels are generally underutilized, despite their growing use in recent years in response to many societal challenges [17]. Factors that may be contributing to this underutilization are the limited empirical evidence concerning the effects of such panels, in addition to their time-consuming, labour-intensive, and costly nature, relative to other, traditional approaches for eliciting citizens’ priorities and preferences, such as surveys. To our knowledge, the use of a citizen panel in the scope of developing an HSPA framework is novel. From the outset, the development of the Irish HSPA framework was anchored in the principles of inclusiveness by considering the engagement of various stakeholders. The engagement of stakeholders was operationalized in the form of panels (including a citizen panel) for the prioritization of HSPA domains and suggestion of performance measures; recurrent meetings with designated DoH and HSE representatives for populating the HSPA framework with indicators based on fit-for-purpose and use criteria [32]; and interviews and workshops with healthcare performance professionals for discussing the health information system in Ireland and validating data sources and indicators.

The engagement of citizens in the early phases of developing the Irish HSPA framework shaped the processes that followed, with the restating of priorities of the citizen panel informing decision-making throughout. For example, during all meetings with designated DoH and HSE representatives for populating the HSPA framework with indicators, the citizen panel’s priorities were restated (including suggestions of performance measures, if need be) and served to inform decision-making on which indicators had the potential to be included in the framework. This process was also followed during the workshops where data sources and indicators were discussed. Even with the benefit of hindsight, it is difficult to envision what the final version of the Irish HSPA framework would have been had the citizen panel not occurred. Therefore, following the principles of inclusiveness in governance [6, 9, 35] and in creating value in healthcare [26, 36], by involving citizens we actively contributed to fostering legitimacy, responsiveness, and accountability of the framework in safeguarding the interests of a broader range of stakeholders [28]. The added value of engaging a citizen panel was also discussed during project meetings with the international advisory board—a consultative group of HSPA experts from different European countries—which welcomed the inclusion of the citizen panel in the process of developing Ireland’s HSPA framework. We consider that including citizens in this process was possible in Ireland given the democratic culture of listening to citizens’ voice during policy-making cycles. In other countries where citizens experience less empowerment or engagement in policy-making processes, using a similar approach to develop an HSPA framework may prove (more) challenging. Based on the successful engagement of citizens in the participatory process of developing the Irish HSPA framework, other initiatives could follow. For example, citizens and other stakeholders could be engaged in discussing the performance of the healthcare system using the framework, or in updating health priorities that should be measured using the HSPA framework. These initiatives have the potential to foster citizen-driven healthcare system performance but call for long-lasting participatory processes. Inspiration could be drawn from the broad participatory process used for developing the Austrian health targets [37]—a guiding framework for public health policy—where an online public consultation was used, and relevant stakeholders were engaged for both development and monitoring purposes.

In general, citizen panellists shared a similar understanding of what a citizen panel involves. This could signal an effect of the citizen brief used to communicate with participants prior to the panel, where we sought to address any information asymmetry by clarifying in lay language what a citizen panel was and presenting the key themes that served as a basis for discussions during the citizen panel workshop. We noted that most of the citizens came well prepared and were fully engaged in the discussions. This was contrary to our initial expectations, where we feared that health literacy levels in Ireland [38] and the participants’ ability to think outside their personal experiences could hinder full participation. Most panellists also considered their participation as part of their civil duty, particularly in terms of representing their fellow citizens. This was aligned with our views on the advantages of using the citizen panel in the development of the framework, where we considered citizen panel participants as proxies for other people as well. Hence, our focus was not on a panellist’s personal experiences per se, but rather about developing a collective perception of the experiences and preferences of the citizen panel as a whole.

### Strengths and limitations

The key strengths of our study are the participatory and inclusive characteristics of the processes of developing the first HSPA framework for Ireland by including three stakeholder panels (including a citizen panel), with the advantage of comparing prioritizations among panels; and the profile of the teams involved during the stages of developing the framework, bringing technical expertise but also having close collaboration with local experts and decision-makers. We recognize that using only one citizen panel limits any ambition to achieve representativeness of Ireland’s population, and that different insights could have been generated had we organized a citizen panel with other participants or multiple citizen panels. Organizing multiple panels, however, would have not been possible due to time and cost constraints; nevertheless, we strove to reach sufficient diversity with the citizens recruited to participate in the panel, relative to the general population of Ireland. Another limitation relates to the difficulty in controlling all aspects regarding the process of recruiting panellists; either having an established database from which a sampling strategy could be employed, or commissioning the recruiting of panellists to a local agency is probably best.

## Conclusions

In the context of Ireland’s *Sláintecare* health reform, strengthening participatory citizenship and end-to-end accountability on the performance of the health system has become a topic of interest to a broader audience, hence the intent of strengthening the voice of citizens as part of shared decision-making in the development of an HSPA framework. Following principles of inclusiveness in creating value in healthcare, citizens’ engagement in the development of an HSPA framework is a crucial contribution for realizing people-centred value-based health systems. The engagement of citizens also contributes to public trust and a sense of health system ownership, of which the importance has only been enforced over the period of the COVID-19 pandemic. Although HSPA is a country-owned process, it builds off of international practices and experiences. Other countries that seek to strengthen value creation in their health systems via the engagement of citizens in HSPA development cycles could draw from these experiences and learnings in Ireland. Future research could expand the use of citizen panels to assess, monitor, and report on the performance of healthcare systems.

## Supplementary Information

**Additional file 1**: Internal and external stakeholder panels.

**Additional file 2**: Citizen panel recruitment scheme and considerations.

**Additional file 3**: Citizen recruitment screening questionnaire.

**Additional file 4**: Citizen brief and workshop programme.

**Additional file 5**: Citizen panel exit questionnaire.

**Additional file 6**: Citizen panel: Summary of key findings.

**Additional file 7**: Visualization of the Irish HSPA framework and its elements.

**Additional file 8**: Citizen panel’s suggestions of performance measures and performance-related topics.

## Data Availability

Data sharing is not applicable to this article as no datasets were generated or analysed during the current study.

## Abbreviations

HSPA: Health system performance assessment
DoH: Department of Health
HSE: Health Service Executive
