# Supplementary material for: Engaging citizens in the development of a health system performance assessment framework: a case study in Ireland
Source: Health Res Policy Syst. 2021 Dec 20;19:148. doi: 10.1186/s12961-021-00798-8 (PMC8685819; doi:10.1186/s12961-021-00798-8)
Supplement: Supplementary file 1 — Additional file 1: Internal and external stakeholder panels. [file 12961_2021_798_MOESM1_ESM.pdf]

Additional file 1
Internal and external stakeholder panels

Session organization

The Internal and External Stakeholder panels (Panel 2 and Panel 3, respectively) were run after the citizen panel (Panel 1), on 31 January 2020, Dublin. Around 30 people participated in Panel 2, and 20 people in Panel 3. The panels were facilitated by the Department of Health (DoH), including selection of participants; moderation was done by the research group from the University of Amsterdam. The Internal Stakeholder panel (Panel 2) accounted for people from the DoH and Health Service Executive (HSE). The External Stakeholder panel (Panel 3) included representatives from health and care services, patient organisations, and academic institutes. One week before the meeting, all participants received via e-mail a brief with supporting information for the workshop, including the agenda for the day. The brief was similar to that distributed among participants in the citizen panel.

Table AF1.1 Session organization of the internal and external stakeholder panels

Task	Brief description	Expected duration
Welcome and introductions	Round of introductions (moderators and participants)	15 mins
Context of developing an HSPA Framework for Ireland	• Inform about the aims of developing an HSPA framework for Ireland<br>• Inform about the project phases and key actors<br>• Brief explanation on what is and what is not relevant for the purpose of measuring a health (care) system's performance<br>• Examples of how other countries approached the development of their HSPA frameworks, including the most frequently used domains	15 mins
Exercise 1: Inventory of domains<br><br>What should be measured and reported on in Ireland?	Participants share their views on key domains that should be featured in the Irish HSPA framework. The moderators will account for how many times a domain is signalled by participants.	45 mins
Exercise 2: Populating domains with indicators<br><br>Discussion on what to measure and report on in Ireland?	Based on the results in exercise 1, participants are invited to write down on coloured sticky notes indicators that should be featured in the Irish HSPA framework.	30 mins
Results from the Citizen Panel & Wrap-up	Moderators share and discuss the results of the prioritisation exercise in the citizen panel. Wrap-up.	15 mins

Domain prioritisation in the internal and external stakeholder panels

The output of the panels resulted in a listing of domains according to the frequency they were mentioned by participants (Figure S1). Both panels yielded similar domains, although prioritised somewhat differently.

Internal Stakeholders (Panel 2)	Number of times mentioned	External Stakeholders (Panel 3)	Number of times mentioned
**1** Accessibility	23	**1** Accessibility	14
**1** Responsiveness	23	**1** Responsiveness	14
**2** Effectiveness	16	**1** Social and financial risk protection	14
**2** Equity	16	**4** Efficiency	10
**2** Social and financial risk protection	16	**4** Health and well-being	10
**2** Coverage	16	**4** People-centeredness	10
**7** Efficiency	15	**7** Equity	9
**8** Health and well-being	13	**7** Coverage	9
**9** Coordination of care	12	**9** Quality of care	8
**9** Continuity of care	12	**10** Effectiveness	6
**11** Quality of care	10	**10** Health workforce	6
**12** People-centeredness	9	**12** Coordination of care	5
**13** Safety	6	**12** Continuity of care	5
**14** Health workforce	1	**14** Safety	4

Structures Processes Health outcomes Outputs Cross-cutting

Figure AF1.1 Prioritization of HSPA domains by the internal and external stakeholders' panels. Color indicates clustering of domains as depicted in the produced HSPA framework for Ireland

Participants from the Internal Stakeholder panel produced 235 sticky notes with performance measures that could be featured in the Irish HSPA framework (50 green sticky notes, meaning these were somewhat important measures; 87 yellow sticky notes, signalling important measures; and 98 red sticky notes, signalling extremely important measures). The External Stakeholder panel produced 128 sticky notes (9 green; 8 yellow; 111 red).
