# Supplementary material for: Engaging citizens in the development of a health system performance assessment framework: a case study in Ireland
Source: Health Res Policy Syst. 2021 Dec 20;19:148. doi: 10.1186/s12961-021-00798-8 (PMC8685819; doi:10.1186/s12961-021-00798-8)
Supplement: Supplementary file 2 — Additional file 2: Citizen panel recruitment scheme and considerations. [file 12961_2021_798_MOESM2_ESM.pdf]

Additional file 2
Citizen panel recruitment scheme and considerations

Recruitment of citizens
Citizens will be recruited for the panel workshop directly by a recruitment company. The required number of citizens is a maximum of 15. Additionally, 5 more participants shall be invited to ensure that the required number of citizens for the panel will be met albeit last minute cancelations or drop-outs.

A definition of a citizen eligible to participate in this panel is that of a lay person, resident of Ireland, who is not directly professionally involved with the health care system and is not a public official.

The sampling strategy aims to achieve a **reasonable diversity** of citizens **considering the sociodemographic characteristics of the population** of Ireland. The recruitment should allow to convene a panel based on the following eight diversity factors:

1. Population distribution across counties of Ireland
2. Sex
3. Age
4. Highest level of education attained
5. Nationality
6. Ethnic or cultural background
7. Health status
8. Religious beliefs

To support a reasonable diversity of citizens further considerations will be made on the aforementioned criteria, based on the official census 2016 data.

1. Population distribution across counties
All four Provinces should be represented, and it is expected that Counties with more inhabitants will be more represented. A reasonable representation of citizens from urban and rural areas is expected.

2. Sex
It is expected for the panel to be well-balanced in terms of men and women (1:1 ratio).

3. Age
All citizens should be 18 years old and over. Although there is no age limit to participation in the panel, special attention should be paid to older age groups with regard to their ability to fully engage with the panel activities and necessary traveling to the meeting. In general, the panel should reflect diversity considering the following age groups:

- young adults (18–25; 25–34);
- adults (35–44; 45–54);
- middle aged adults (55–64);
- older adults (65 and over).

Crosstab information regarding Census data on county by age group provide further insight on a possible composition of the citizen panel (Table AF1.1).

Table AF2.1 Target age mapping by location

Province	County	Target recruitment	Age group					
			**18–25**	**25–34**	**35–44**	**45–54**	**55–64**	**65+**
**Connacht** Western coast	Galway	1					1	
	Mayo	1	1					
**Leinster** Eastern coast	Dublin	5		1	1	1	1	1
	Kildare	1			1			
	Meath	1				1		
	Wexford	1				1		
	Wicklow	1		1				
	Louth	1			1			
	Kilkenny	1		1				
**Munster** Southwest coast	Cork	2	1					1
	Limerick	1					1	
	Tipperary	1				1		
	Kerry	1			1			
**Ulster**	Cavan	1			1			
	Monaghan	1		1				
		20	2	4	5	4	3	2

4. Highest level of education attained

Highest level of education attained could be divided into three categories: primary or less, secondary (included lower and upper secondary, vocational and advanced certificate), and tertiary. Considering the population aged 20 and over, the distribution of highest level of education attained to be reflected in the composition of the citizen panel is as follows:

- **Primary** – 11%; age groups most represented are 65+ (4.5%) and 55-64 (2.8%).
- **Secondary** – 51%; age groups most represented are 45-54 (12.3%), 35-44 (11.4%) and 55-64 (10.2%).
- **Tertiary** – 38%; age groups most represented are 35-44 (11.8%) and 25-34 (10.1%).

Hence, half of the citizen panel (10 citizens) should have concluded secondary education, 8 citizens in the panel should have concluded tertiary education, and the remainder 2 should have concluded primary education.

5. Nationality

A reflection of the multiplicities of nationalities in the Irish society should be to some extent visible in the panel. As the official proportion of non-Irish nationals is 12%, 2 panelists should be non-Irish. Ideally, these panelists should represent the following nationalities: 1 from the United Kingdom and the other preferably from Poland, Romania or Brazil. This accounts for the countries of origin most prevalent in Ireland.

6. Ethnic or cultural background

Around 90% of the citizens of Ireland are white; 3% did not state ethic or cultural background. Based on those data, we expect that 1 of the panelists to be from a different ethnic or cultural background (e.g., Chinese, other Asian, Black).

7. Health status

According to the Irish Health Survey 2015, 32% of the population have a long-standing illness or health problem. Hence, we expect that about 6 citizens to participate in the panel to have a long-standing illness.

8. Religious believes

Considering the distribution of the population by religious grouping it is expected that most panelist will be Roman Catholic. Other religious believes (or none) should be represented as well (e.g., Church of Ireland, Islamic).

Limitation to the recruitment of citizens

Citizens must show a sufficient level of proficiency with the English language (speaking, listening, reading). The meeting will occur in the city of Dublin, which may limit the ability of citizens to travel to the meeting location and back to their hometown. A rough estimation for travel time is as follows (Table AF1.2).

Table AF2.2 Travel time to Dublin from counties across Ireland

**Province**	**County**	**Travelling time**
**Connacht** Western coast	Galway	2h30 [1]
	Mayo	3h00 [2]
**Leinster** Eastern coast	Dublin	0h30 [1]
	Kildare	0h45 [1]
	Meath	0h45 [2]
	Wexford	2h00 [1]
	Wicklow	1h20 [1]
	Louth	1h00 [2]
	Kilkenny	1h30 [1]
**Munster** Southwest coast	Cork	3h00 [1]
	Limerick	2h45 [1]
	Tipperary	2h00 [2]
	Kerry	3h30 [2]
**Ulster**	Cavan	2h00 [1]
	Monaghan	2h00 [1]

[1]Travel by train/bus up to 1 transfer

[2] Travel by car
