# Supplementary material for: Engaging citizens in the development of a health system performance assessment framework: a case study in Ireland
Source: Health Res Policy Syst. 2021 Dec 20;19:148. doi: 10.1186/s12961-021-00798-8 (PMC8685819; doi:10.1186/s12961-021-00798-8)
Supplement: Supplementary file 3 — Additional file 3: Citizen recruitment screening questionnaire. [file 12961_2021_798_MOESM3_ESM.pdf]

Additional file 3
Citizen recruitment screening questionnaire

The Irish government is committed to its ten-year health plan Sláintecare and understanding how well the health system performs is important to know how it is working and for finding ways to improve it. We are aiming to bring together a small group of Irish citizens for a one-time discussion on the topic of 'Measuring and reporting on the performance of Ireland's health system to the public'.

No prior knowledge on the health system is required. You will be offered an incentive for your attendance. The panel will be held over one day for a total of 4 hours. It will begin at 11am on Saturday 7th December and is expected to close no later than 15:00. Approximately 1 hour of preparatory reading is required one week in advance of the event to review the citizen panel brief. If you would like to share your views on what you, as a citizen of Ireland want from our health system, then please answer the following questions to express your interest.

Participants are expected to have a good working knowledge of written and spoken English.

Q.1 Are you available to take part in this project in a central Dublin location on Saturday 7th December, from 11am to 3pm?

Yes 1
No 2

Close if Q.1/No

Q.2 How do you intend to travel to the Dublin venue?

Walk 1
Bike 2
Car/motorcycle 3
Bus 4
Train 5
DART 6
Luas 7
Taxi 8
Get a lift 9
Other (please specify) 10

Check Cost with Client

Q.3 Do work in any of the following areas?

READ OUT
Advertising 1
Public Office 2
Healthcare 3
Marketing 4
Market Research 5
None of the above 6

Close if Q3 1-5 ticked

Q.4	What is your gender?	Male .... 1<br>Female .... 2<br>Other .... 3<br><br>**Check Quota Targets**
Q.5	What county do you live in?	Dublin .... 1<br>Cork .... 2<br>Cavan .... 3<br>Galway .... 4<br>Tipperary .... 5<br>Kildare .... 6<br>Kilkenny .... 7<br>Kerry .... 8<br>Limerick .... 9<br>Louth .... 10<br>Mayo .... 11<br>Monaghan .... 12<br>Tipperary .... 13<br>Wexford .... 14<br>Wicklow .... 15<br>Other (please specify) .... 16<br><br>**Check Quota Targets**
Q.6	What is your nationality?	Irish .... 1<br>English .... 2<br>Polish .... 3<br>Asian .... 4<br>Indian .... 5<br>Romanian .... 6<br>Brazillian .... 7<br>Other (please specify)........ 8<br><br>**Check Quota Targets**
Q.7	What is your ethnic or cultural background?	Caucasian .... 1<br>Asian .... 2<br>Black/African .... 3<br>Hispanic/Latin .... 4<br>Other (please specify) .... 5<br><br>**Check Quota Targets**

Q.8	What is your current religion, if any?	Roman Catholic .... 1<br>Church of Ireland .... 2<br>Jewish .... 3<br>Muslim .... 4<br>Atheist .... 5<br>Prefer not to say .... 6<br>Other (please specify) 7
Q.9	How would you evaluate your overall health? Would you say you are:	In good physical health (no illness or disabilities) …………………………………………………..………..1<br>Mildly physically impaired (minor illness or disabilities) ………………………………..……… 2<br>Moderately physically impaired (requires substantial treatment) …..................................... 3<br>Severely physically impaired (requires extensive treatment) ……………………………………… 4<br><br>**Check Quota Targets**
Q.10	What is your highest level of education?	Junior Certificate (or equivalent) .... 1<br>Leaving Certificate (or equivalent) .... 2<br>Certificate/Diploma .... 3<br>BA/BSc .... 4<br>Graduate Diploma/Masters .... 5<br>PhD .... 6<br>No education qualification .... 7<br>Other (please specify) .... 8<br><br>**Check Quota Targets**

Interviewer: Thank respondent, record respondent's name, age (Check Quota Targets), email and phone number.
