# Supplementary material for: Engaging citizens in the development of a health system performance assessment framework: a case study in Ireland
Source: Health Res Policy Syst. 2021 Dec 20;19:148. doi: 10.1186/s12961-021-00798-8 (PMC8685819; doi:10.1186/s12961-021-00798-8)
Supplement: Supplementary file 4 — Additional file 4: Citizen brief and workshop programme. [file 12961_2021_798_MOESM4_ESM.pdf]

Additional file 4
Citizen brief and workshop programme

Citizen Panel Brief

What to know, and how to be informed about how Ireland's health system is doing

---

11:00–15:00, Saturday 7 December 2019

Skylon Hotel, 27 Drumcondra Rd Upper
Drumcondra, Dublin, D09 V1Y2, Ireland

Research team
This brief has been prepared by (alpha order) Erica Barbazza, Óscar Brito Fernandes, Niek Klazinga and Dionne Kringos of the Amsterdam University Medical Centre, location Academic Medical Centre of the University of Amsterdam.

Funding
This citizen brief and the citizen panel is funded by the European Commission Structural Reform Support Service project SRSS/C2019/046 – "Performance accountability for the Irish health system." The document reflects on the authors' views and the European Commission is not responsible for any use that may be made of the information that it contains.

About this event

What is a citizen panel?

A citizen panel is a way to get the public's input on high-priority issues. The process is used in a number of countries to help policy-makers make decisions. Panels typically bring together citizens from all walks of life. By participating in a citizen panel, citizens like yourself are able to express your ideas and experiences on a specific issue for decisions that consider public opinion.

About the organizers

The Health Services and Systems Research Unit of the Academic Medical Centre at the University of Amsterdam studies the effective use of health care performance data to improve services, and ultimately, health outcomes. We work locally, nationally and internationally to bridge our research with decision-makers and support health care reforms. Our research unit was contracted by the European Commission[1] to carry out this citizen panel and other activities related to the project "Performance accountability for the Irish health system."

About this brief

This brief was produced by the Academic Medical Centre of the University of Amsterdam to serve as a basis for discussions during the citizen panel on measuring and reporting on the performance of Ireland's health system.

Output of this panel

All participants will receive a summary document on the citizen panel. In early 2020, this document will also be shared at a workshop with representatives of the Department of Health and other key stakeholders. The feedback from the citizen panel will be taken into consideration together with scientific evidence for making decisions about what to measure and report on in Ireland about the health system's performance.

---

[1] For more information about the Structural Reform Support Service of the European Commission and to learn more about other projects funded by the Programme, visit: https://ec.europa.eu/info/departments/structural-reform-support-service_en

Background: why is it important to know how the health system is doing?

There is a need for a public conversation about what we need to know about the health system in Ireland in order to make it work better.

Why does this matter in Ireland right now?

It is an important time to discuss what information policy-makers, health care providers and citizens would like to have about how the health system is doing (in other words, how it is performing) for a number of reasons. For example:

- **Ireland aims to improve the health care services people receive by its new programme, called 'Sláintecare'**.
- **At the moment, it is difficult to know how the health system is doing and where it needs to be changed or kept the same**. This is partly because we are not yet collecting and publicly sharing the information people find important.
- **Developing a method to collect the right information on the health system in Ireland has been prioritized** and a number of activities, like this citizen panel, have been organized with the funding support of the European Commission to help the process.

Why is it important to know how the health system is doing (performing)?

We find four key reasons why it is important to know how the health system in Ireland is doing.

1. **To identify things that need improvement, change or attention** so that the health system will work better to meet the needs and expectations of patients and citizens.
2. **To be open and take responsibility** on how the health system is doing.
3. **To see over time how the health system is changing**, since health needs and the expectations of citizens may also change over time.
4. **To plan and organize the different activities of the health system** so that different providers like nurses, general practitioners or hospital doctors will work better to care for patients.

The problem: why is it difficult to decide on what we want to know about the health system?

Collecting information on how the health system is doing is complex. This is because of a number of factors.

1. **There are also things outside the health system that influence health**

   One key challenge to collecting information on how the health system is doing, is deciding what to measure. This is partly because there are many things that take place outside of the health system that may influence a person's health and wellbeing. For example, the education system and the environment.

2. **There is no "right way" to collect and share information about how the health system is doing**

   What type of information to collect and *how* to share this information needs to be decided in each country on its own. There is a lot that can be learned from other countries or from international organizations like the World Health Organization. However, in the end, it is up to Ireland to decide what information will be collected, used and shared on the Irish health system.

3. **Access to good, trustworthy data is important but also difficult**

   Sometimes what we want to know is difficult to get because we do not have the right information available, or it is too expensive to get those data. Therefore, it is important to decide what is prioritized for measuring in the future.

Topics for discussion: what do you want to know about your health system, and in what way do you want to receive this information?

We are seeking public input on what is most important for you to know about how the Irish health system is doing and how you want to receive this information.

To judge how the health system is doing, information needs to be collected on a set of priority topics. The selection of such topics is an important decision. These topics are typically called *domains.* For each domain, information on more than one aspect of the health system could be collected.

We have summarized the most commonly used *domains* by other countries. We included a short explanation of what it means and an example of the type of information that can fall under that domain.

Domain	What does it mean? How do we measure it?
**Health and well-being**	The state of health and well-being reported by major themes related to health at large. For example, how long people are expected to live (life expectancy).
**Equity**	Delivering health care services that do not differ according to personal characteristics such as gender, race, ethnicity, geographical location or socioeconomic status. For example, the differences between counties in life expectancy.
**Accessibility**	The ability for individuals to easily reach health services in terms of location, time and convenience. For example, the number of individuals waiting for more than 120 days for a specific health care appointment.
**Quality of care**	The degree to which health services increase the likelihood of desired health outcomes and are consistent with current professional knowledge. For example, the number of births by caesarean section instead of natural deliveries.
**Social and financial risk protection**	The extent to which people can pay for the services needed without undue hardship. For example, how much people pay out-of-pocket payments for health care services or medications.
**Coverage**	The extent to which people in need of health care actually receive it. For example, the number of children immunized (vaccinated) to prevent childhood diseases.
**Safety**	Health care that minimizes risks and harm to patients, such as avoiding accidents, hospital complications, preventable injuries and reducing medical errors. For example, the number of slip and falls that occur in long-term care facilities.
**Responsiveness**	The ability of the health system to meet the needs and expectations of citizens. For example, the overall satisfaction of users with the health care system.

Domain	What does it mean? How do we measure it?
**Efficiency**	The delivery of health care in ways that maximizes resource use and avoids waste. For example, the number of generic (no-name) drugs rather than brand names prescribed by doctors.
**Effectiveness**	Health care services delivered based on scientific knowledge and the best available evidence. For example, the number of people that are go back to the hospital within 30 days of being hospitalized (readmissions).
**People-centred**	Care that takes into account the preferences and needs of citizens. For example, doctors taking into consideration the treatment wishes or preferences of patients.
**Continuity of care**	The delivery of care over time so a patient experiences a continuous caring relationship with their regular health care provider and undisrupted treatment. For example, a doctor's follow-up on a patient's medication for people with chronic conditions.
**Coordination of care**	The deliberate organization of a patient's care between two or more health care providers (e.g. a specialist in a hospital and their regular general practitioner) to ensure the best delivery of health care services. For example, the time taken for a patient's to be referred back to their general practitioner after being in hospital.

Glossary of key terms

**Health**	The state of complete physical, mental and social well-being, and not only the absence of disease. Measuring the state of health includes tracking causes and numbers of death, disease, disability as well as well-being, such as quality of life.
**Health services**	Any service with the aim of improving health or the diagnosis, treatment and rehabilitation of sick people.
**Health system**	All organizations, people and actions whose *main aim* is to promote, restore or maintain health. This may include actions that target the population as a whole. The health system connects with other systems, like social care.
**Health system functions or "building blocks"**	Health systems are made up of a core set of parts that have been described as "building blocks" or functions. These often include: *delivering services* (e.g. primary care, specialized services); the *health workforce* (e.g. nurses, family doctors, specialists); *health information* (e.g. medical records, patient registries); *medical products and technologies* (e.g. prescription medication, diagnostic tools); *financing* (e.g. pooling of funds, health care spending); and *stewardship* (e.g. planning health policies, regulating the health system).
**Health system performance**	The extent to which a health system carries out its main functions to achieve defined goals. Where these functions refer to the above (delivery of services, health workforce, health information, medical products and technologies, financing and stewardship).
**Health system performance assessment**	The assessment of the health system as a whole, using clusters of *domains* and a limited number of *indicators* to link outcomes with the different functions of the health system. It is a country specific process, meaning what is assessed should be linked to the goals of specific policies or plans. Assessments are typically run regularly, for example, once every two years.
**Domain**	Each framework defines specific performance domains (sometimes called dimensions) as the specific elements deemed important to assess the health system's performance.
**Indicator**	A well-defined performance measurement metric that is used for monitoring. A range of indicators are needed to assess the performance of the health system. Examples of indicators include average hospital stay (the amount of time a patient stays in the hospital on each visit), waiting time (how long it takes to see a particular health care practitioner or to receive a specific service).

Performance Accountability
for the Irish Health System
European Commission Structural Reform Support Service
NºSRSS/C2019/046

Citizen panel: Measuring and reporting on the performance of Ireland's health system

11:00–15:00, Saturday 7th December 2019
Skylon Hotel 27 Drumcondra Rd Upper, Drumcondra, Dublin D09 V1Y2, Ireland

Provisional Programme

Moderators
Dionne Kringos, Erica Barbazza and Óscar Brito Fernandes, University of Amsterdam

10:30–11:00	Registration opens
	You will receive a printed version of the Citizen Brief and programme. You will also receive a letter of consent for your review and signature. Tea and coffee will be available.
**11:00–11:15**	**Welcome and introductions**
	The moderators will begin with a round of introductions. They will present the aims and objectives of the day and the programme. There will be time to ask any questions you may have.
**11:15–11:45**	**Discussion on the context and challenges**
	The moderators will shortly present the following three topics. There will be time to discuss and ask any questions you may have.<br>• What would you like to know about how the Irish health system is doing?<br>• Why is measuring and reporting on the how the health system is doing important?<br>• What are the challenges to measure and report on how the health system is doing?
**11:45–12:30**	**What should be measured and reported on in Ireland?**
	The moderators will give examples of common elements used to measure and report on health systems. A group exercise will follow where you will have the chance to consider what is most important to you to measure and report on in Ireland.
12:30–13:00	Break and light lunch (sandwiches) will be provided
**13:00–14:45**	**Discussion on what to measure and report on in Ireland**
	In this session, the moderators will summarize the main elements the group found important. As a group, the following two topics will be discussed:<br>• What should be prioritized to measure and publicly report on in Ireland?<br>• How can performance information on the Irish health system best be reported?
**14:45–15:00**	**Summary and wrap-up questionnaire**
	The moderators will summarize the key messages of the day. A short questionnaire will be circulated for your feedback.
15:00	Closing and admin processes
	A representative from Focus Group Ireland will be present to coordinate reimbursement and travel expenses. They can also address any logistic questions you may have.
