# Supplementary material for: Engaging citizens in the development of a health system performance assessment framework: a case study in Ireland
Source: Health Res Policy Syst. 2021 Dec 20;19:148. doi: 10.1186/s12961-021-00798-8 (PMC8685819; doi:10.1186/s12961-021-00798-8)
Supplement: Supplementary file 5 — Additional file 5: Citizen panel exit questionnaire. [file 12961_2021_798_MOESM5_ESM.pdf]

**Additional file 5**
**Citizen panel exit questionnaire**

# Wrap-up questionnaire

---

Thank you for your participation in today's citizen panel on *Measuring and reporting on the performance of Ireland's health system*. We would like to hear about your experience in taking part in this event, for which we kindly ask you to answer the following questions. Your responses are anonymous and will be used only by the research team.

---

**1. How would you describe your experience in taking part in today's citizen panel?**

**2. To what extent did you feel that you were sufficiently prepared to take part in the panel?**

**3. Using your own words, please complete the following sentence:**

**"Citizen panels are …**

**4. Are there any other comments regarding your experience, the organization of the event or other topics that you would like to share with the organizers?**

**Thank you** for your efforts and participation!
