# Supplementary material for: Engaging citizens in the development of a health system performance assessment framework: a case study in Ireland
Source: Health Res Policy Syst. 2021 Dec 20;19:148. doi: 10.1186/s12961-021-00798-8 (PMC8685819; doi:10.1186/s12961-021-00798-8)
Supplement: Supplementary file 6 — Additional file 6: Citizen panel: Summary of key findings. [file 12961_2021_798_MOESM6_ESM.pdf]

Additional file 6
Citizen panel: Summary of key findings

Citizen Panel Summary

Citizens’ viewpoint on what they want to be informed on regarding the performance of their health care system

---

The citizen panel occurred on December 7th, 2019

Dublin, Ireland

Funding
The citizen panel and related materials, including this summary, were funded by the European Commission Structural Reform Support Service project SRSS/C2019/046–“Performance accountability for the Irish health system.” The document reflects only the authors’ views and the European Commission is not responsible for any use that may be made of the information that it contains.

About convening a citizen panel

What is a citizen panel?

A citizen panel is a way to get the public's input on high-priority issues. The process is used in a number of countries to inform the decisions of policy-makers and other key-stakeholders. Panels typically bring together citizens from all walks of life. By participating in a citizen panel, citizens are able to express their ideas and experiences on a specific issue for decisions that consider public opinion.

About the research team

The Health Services and Systems Research Unit of the Academic Medical Centre at the University of Amsterdam studies the effective use of health care performance data to improve services, and ultimately, health outcomes. We work locally, nationally and internationally to bridge our research with decision-makers and support health care reforms. Our research unit was contracted by the European Commission[1] to carry out and report on a citizen panel and other activities related to the project "Performance accountability for the Irish health system."

About this summary

On the 7th of December 2019, the research team convened and moderated a citizen panel on measuring and reporting on the performance of Ireland's health care system. This summary aims to highlight the key views and common experiences of panel participants on what is most important for them to know about how the Irish health care system performs and also how they wish this information is reported on and disseminated[2]. This summary of the day's discussions will serve as a background document for a stakeholder meeting on measuring and reporting on the performance of Ireland's health system that will take place in January 2020.

[1] For more information about the Structural Reform Support Service of the European Commission and to learn more about other projects funded by the Programme, visit: https://ec.europa.eu/info/departments/structural-reform-support-service_en

[2] All citizens that have participated in the panel have received a summary document on the citizen panel discussions.

Panel participants: how were they recruited and what was their profile?

The goal of the recruitment was to achieve a reasonable diversity of citizens, considering the sociodemographic characteristics of the population of Ireland.

How did we conduct the recruitment of citizens?

Citizens were recruited by a third-party Dublin-based company with years of expertise in the recruitment of citizens for similar meetings. A definition of a citizen eligible to participate in the panel was that of a lay person, citizen of Ireland, who is not directly professionally involved with the health care system and is not a public official.

The selection strategy aimed to achieve a reasonable diversity of citizens considering sociodemographic characteristics of the population of Ireland[3]. Diversity factors taken into consideration were, for example, sex, age group, highest level of education attained, nationality, ethnic/cultural background and health status. Participants were compensated for their travel and a small stipend for their time.

What was the profile of panel participants?

A total of 15 participants of different walks of life participated in the citizen panel; 7 were men and 8 women.

1. **The age group distribution** was as follows: 18—24 (n=2); 25—34 (n=4); 35—44 (n=1); 45—54 (n=4); 55—64 (n=1) and; 65 and over years (n=3).

2. **The highest educational level** for one participant was the Junior Certificate; for 27% of the participants (n=4) was the Leaving Certificate; 3 participants had a Certificate/Diploma; 40% (n=6) had a Bachelor diploma and one participant had a Graduate/Master's certification.

3. **The nationality of most respondents (80%)** were Irish, one participant was Irish with Asian background and two other participants were from European countries (Croatia and Italy) residing in Ireland.

4. **The participants' health status** was as follows: 27% had a persistent or permanent health condition, whereas remaining participants were in good physical health.

[3] To support a reasonable diversity of citizens we based our considerations regarding the distribution of the sample following the Census 2016 data.

Panel organization and structure

1. **Citizen brief**

Prior to participating in the citizen panel, all participants received a Citizen Brief. This document provided general information regarding the measurement and reporting on the performance of a health care system. It was structured in three sections:

- **Background** information on why it is important to know how the health care system is performing;
- A description of the **Problem** of why it is difficult to decide on what kind of performance information is desirable about the health care system;
- Starter-questions on the **Discussion** topics for the panel, focusing on what citizens want to know about the performance of their health care system and which way they would prefer to receive that information.

2. **Citizen panel activities**

The activities of the panel were structure in three main blocks:

- **Synthesizing the reasons to convene the citizen panel** in light of the current efforts to measure and report on the health care system's performance;

Following a round of introductions, the citizens had the opportunity to briefly share their overall satisfaction with the health care system.

- Collect information on the **citizens' experiences and preferences on what information should be measured** and reported on Ireland;

During a walk-through of the most frequent domains featured in health care system performance assessment frameworks, citizens manifested their interest on specific topics/measures using a colored scheme of sticky notes which reflected the degree of importance of that topic/measure.

- **Prioritizing what to measure and report on the performance of the health care system**, as well as identifying approaches and channels whereby information on the health care system could be reported back to citizens.

The heat mapping produced with the three colored sticky notes to identify topics/measures that were important to the citizens, was discussed in terms of clarifying priorities and identifying how those measures should be reported on to citizens and by which channels.

Discussing the topic: overall perception on how the health system is performing–what to measure and report on the performance of the health care system, and through what channels should that information be received by citizens?

Key findings of the panel discussion, where all participants stated to be interested in more information on how the health care system is performing.

Overall perception on the performance of the health care system

The participants' interactions and experiences with the health care system varied greatly. The following synthesizes their perceptions, experiences and satisfaction with regards to the performance of the health care system:

- All participants had great trust in the level of experience and skills of the health care workforce;

- Half of the participants (n=8) had private insurance schemes in place because of their distrust of the health care system and a described necessity to ensure reliable access to services. The latter relates to a generalized perception among the participants of an increasing gap between the public and private schemes and by increasing out-of-pocket payments that are requested from citizens;

- Most participants highlighted negative experiences related to: i) the fact that they had to be their own information carrier because of several limitations in regards to data exchange between information systems; ii) inefficiencies of processes of care (especially at a hospital setting) and; iii) the perception that services are generally understaffed;

- Most respondents recognized the A&E as the entry point to the health care system, partly explained by the limited scope of practice of general practitioners (GPs) (e.g. inability to perform routine examination procedures such as an X-ray);

- Hospital capacity (number of beds) is very limited because of the incapacity of the system to properly handle hospital admissions;

- There is little investment on key areas such as public health services, maternal and child health prior birth and mental health;

- The experiences of care with GPs are generally more positive than those with doctors at hospital;

- A key challenge relates to the long waiting times to get an appointment and at a doctor's office (both GPs and specialist care). This was perceived as a greater problem if one considers the case of older people;

- It is somewhat difficult to access and navigate through the health care system, especially if one is an expat.

Where did participants access information on the health care system?

Common ground among participants was that their perception of the health care system and its challenges are much influenced by what is broadcasted by the media and central in the political debate. Regarding the latter, some citizens mentioned that the discussions are often centered around waiting time issues.

Other sources of information are websites (e.g. Ireland's Health Service Executive [HSE] website) and reports. However, some participants highlighted to feel overwhelmed by the quantity of information available, that it is often difficult to interpret, especially when statistics are presented.

Preferences and priorities about which information should be reported to citizens

Methodological approach

To collect information on the preferences of participants on what to measure regarding the performance of the health care system, we proceeded as follows: first, the moderators briefly summarized the most commonly used *domains* by other countries to report on the performance of their health care systems[4]. After the presentation of a domain, respondents were asked to write down on sticky notes what measures/topics were of concern to them, regarding that domain.

The sticky notes were of three colors (red, yellow and green) to capture the degree of importance of that measure to the participant (extremely important, important, somewhat important, respectively).

[4] Given the limited time of the panel, where needed domains were grouped. Domains presented and discussed with citizens were as follows: health and well-being; access and coverage of care; equity; social and financial risk protection; quality and safety; people-centeredness; efficiency and effectiveness; continuity and coordination.

After a walk-through of all domains, the moderators grouped the sticky notes on a whiteboard in cohesive groups[5], producing a heat map of sticky notes under each group. A discussion about which measures and domains were of top priority to citizens followed.

Key discussions with citizens on their preferences and priorities

- In total, citizens wrote down on 249 sticky notes: 153 were marked as extremely important to citizens, 79 were important and 17 were somewhat important.

- ACCESS (e.g. waiting times in general and across specialties, differences between counties) were of central importance to the panel at-large. Other measures under this domain included:
  - distribution of practices locally, the rates (fees) charged by those practices and reviews of other citizens that have attended that practice;
  - list of healthy lifestyle support services that are provided locally;
  - average length of stay in hospitals;
  - number of doctors per population;
  - clarity on waiting lists between public and private systems;
  - waiting time to get a procedure before applying for cross-border care and;
  - clearer information on how to navigate through the system with emphasis to different modes of access (e.g. by phone, online, out-of-hours access).

- Another central domain was that of EQUITY, with particular emphasis to differences between the public and private systems, as the panel agrees on that everyone should have access to proper care, regardless of their ability to afford it.

- Another key domain of citizens' concern was that of SOCIAL AND FINANCIAL PROTECTION. Most participants recognized a lack of transparency of potential costs of care at point-of-entry.
  - For example, GPs fees were identified as very high and with large variation across the country (lack of standardization). Also, information on prices is often very limited and not displayed in a clear manner.

- In the following table we provide a list of key measures for which citizens would like reporting on or recognize that noticeable improvements are needed.

Domain	Suggested measures and improvements
**Efficiency**	• Avoid cancellation of appointments within 24hrs of the date;<br>• Faster reimbursement whenever one gets care abroad, under the cross-border health care directive;<br>• Improvements for navigating within the health care system;<br>• Availability of counselling and support services (e.g. healthy lifestyle education, maternal care) at large;

[5] Groups were based on the domains presented to the panel. Given that the boundaries of some domains are not clear, overlapping of sticky notes occurred. We considered this not to be a problem because our focus was on the content of the sticky note and less on the grouping strategy.

	• A more trustworthy triage system at hospitals.
**People-centeredness**	• Adequacy of the time a doctor spends with the patient;<br>• Mitigate the language barrier in conversation with foreign doctors and the patients;<br>• Greater home care after hospital discharge policies;<br>• Greater involvement of the patient in decision-making;<br>• Clearer information on alternative treatments also suitable to a person's condition.
**Quality of care**	The group shared that, in general, they trust the quality of care provided by their GP practices, hence suggesting not to need much information on their performance. This is partly because of long-lasting relationships and because information is shared among individuals within a community. On the other hand, participants agreed about the importance of having more information available on the performance of hospitals because of a lack of trust on their practices and quality.
**Well-being**	Some participants suggested it of importance to be informed on key measures of different policies that are in place nation-wide such as survival after cancer diagnosis and treatment or the burden of mental health conditions.

Key findings of a post-panel analysis on citizens' preferences and priorities

- A subsequent analysis of the distribution of the sticky notes across domains suggested that people-centeredness, equity, coordination of care, coverage and health and well-being were the domains where respondents identified measures as "extremely important" to be reported on. The following table provides the full distribution of the sticky notes across domains, ordered by degree of importance.

	Degree of importance			
**Domain**	Extremely important	Important	Somewhat important	Total
1. People-centeredness	32	15	0	47
2. Coordination of care	17	14	0	31
3. Coverage	17	10	2	29
4. Health and well-being	16	6	5	27
5. Equity	20	3	1	24
6. Social and financial risk protection	11	6	1	18
7. Accessibility	8	6	3	17
8. Responsiveness	7	7	1	15
9. Safety	7	4	2	13
10. Quality of care	7	2	1	10
11. Efficiency	6	1	0	7
12. Continuity of care	4	2	0	6
13. Effectiveness	1	3	1	5
Total	153	79	17	249

- Alternatively, mindful that many of the topics written down by the citizens could be placed under more than one domain, for each sticky note we allocated up to four deemed domains. Under these conditions, the domains most frequent and with higher number of "extremely important" sticky notes ranked as follows:
    1. People-centeredness;
    2. Coordination of care;
    3. Coverage;
    4. Equity;
    5. Accessibility;
    6. Social and financial risk protection;
    7. Responsiveness.

Citizens' preferences on how the performance of the health care system should be reported on

- The panel had a generalized preference for a two-tier approach to report on the performance of the health care system:

    1. A **tailored website** with concise and lay-language information where the most relevant key measures should be made available.

        - Key features:

            - Data disaggregation at the community-level, with urban/rural divide and composite measures at a regional-level;

            - Information of available services at the community-level;

            - Real-time data on key measures such as waiting times.

    2. A **stand-alone yearly report** disseminated across key media channels (including social media).

- General characteristics of both approaches highlighted by the participants were:

    - The reporting on the performance of the health care system should enable accountability across actors;

    - People would have greater trust in the information reported if they knew that the analysis were conducted or reviewed by an independent body (e.g. ombudsman, public advocate or health commissioner);

    - Graphs reporting on the evolution of key performance measures should be clear and contrast with the results of previous years (time trend);

    - Overall, there was little interest on reporting the performance of the health care system at the national-level, with exception to general health and well-being measures;

    - International comparisons were of little interest to most participants. If these comparisons are to happen, comparators of interest were reported as Finland, Estonia and European Union averages.
