# Supplementary material for: Engaging citizens in the development of a health system performance assessment framework: a case study in Ireland
Source: Health Res Policy Syst. 2021 Dec 20;19:148. doi: 10.1186/s12961-021-00798-8 (PMC8685819; doi:10.1186/s12961-021-00798-8)
Supplement: Supplementary file 7 — Additional file 7: Visualization of the Irish HSPA framework and its elements. [file 12961_2021_798_MOESM7_ESM.pdf]

Additional file 6
Visualization of the Irish HSPA framework and its elements

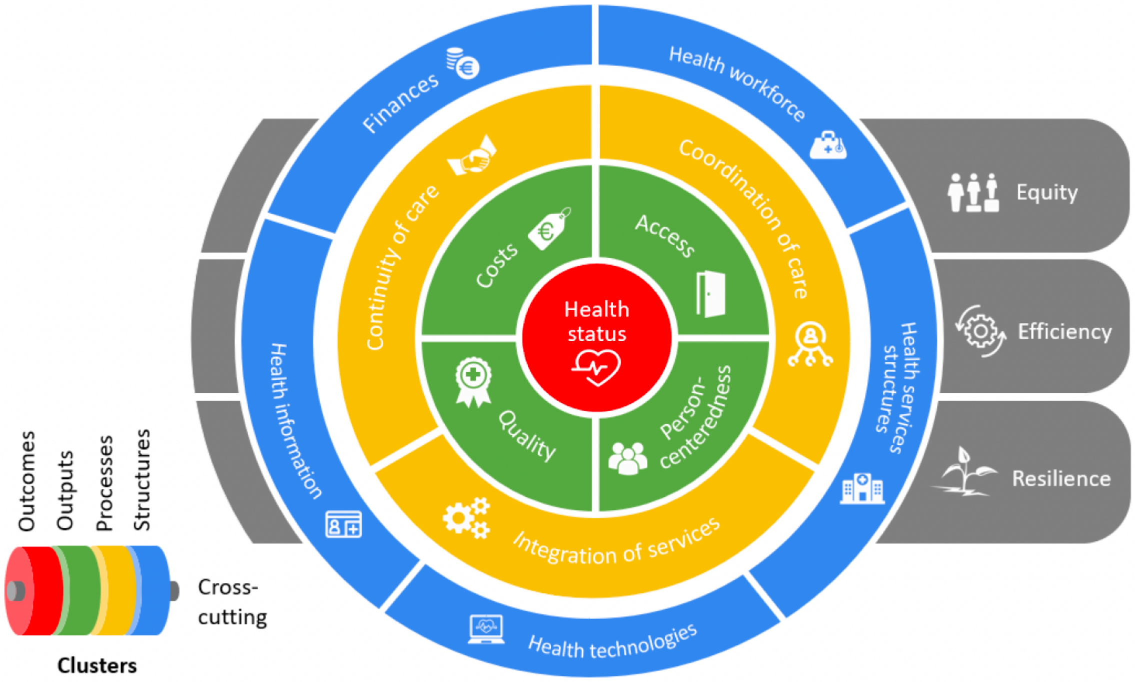

Figure AF6.1 Graphical display of the HSPA framework for Ireland

Table AF6.2 Overview of the HSPA framework for Ireland

Clusters	Outcomes	Outputs	Process	Structures	Cross-cutting	Totals*
Domain	1	4	3	5	3	16
Sub-domain	5	9	6	8	8	36
Approx. features	13	19	5	6	6	49
Approx. indicators	35	95	30	70	30	260

Table AF6.3 Overview of clusters, domains, sub-domains, and features

Cluster	Domain	Sub-domain	Feature
Outcomes	Health status	Self-reported health	Self-reported health status
		Disability	Self-reported disability
			Types of disability
		Morbidity	Burden of disease
			Select types of morbidity
			Multi-morbidity
		Mortality	Life expectancy
			Avoidable mortality
			Causes of mortality
			Healthy life expectancy
		Risk factors	Overvweight/obesity
			Lifestyle/environment
			Health literacy
Outputs	Access	Affordability	Social protection
		Availability	Availability
	Person-centredness	Patient-reported experiences	PREMs
			Satisfaction
		Patient-reported outcomes	PROMs
		Carer experiences	Experience
		Staff experiences	Experience
	Quality	Clinical effectiveness	Cardiovascular and diabetes
			Cancer
			Chronic conditions and ambulatory/primary care sensitive conditions
			Mental health
			Infectious diseases
			Prescribing
		Safety	Medication
			Patient accidents
			Clinical process/procedure
			Health care associated infection
			Resources/Organizational management
	Costs	Costs	Expenditure
Process	Coordination	Self-reported health	Patient
			Carers
			Staff
		Transition management	Follow-up
			Discharge
	Integration	Care delivery	
		Readmission	
	Continuity	Informational	
		Relational	
Structures	Health workforce	Workforce capacity planning	Capacity
			Migration
	Health services structures	Infrastructure	Facilities
			Beds
	Health technologies	Use and uptake of technologies	Technology capacity
			Technology accessibility
			technology diffusion
	Health information	Registries	
		Digitalization	
	Finances	Expenditure	
		Reimbursement mechanisms	
		Investment in R&D	
Cross-cutting	Equity	Population groups	
		Geographic	
	Efficiency	Short-term	Waiting times
		Mid-term	Out of hours care
		Long-term	
	Resilience	Motivated and well-supported workforce	
		Health worker absenteeism	
		Capacity to scale-up/down	
